# Using systems thinking and the Intervention Level Framework to analyse public health planning for complex problems: Otitis media in Aboriginal and Torres Strait Islander children

**DOI:** 10.1371/journal.pone.0194275

**Published:** 2018-03-21

**Authors:** Jo Durham, Lisa Schubert, Lisa Vaughan, Cameron D. Willis

**Affiliations:** 1 Faculty of Medicine, School of Public Health, Herston Road, The University of Queensland, Queensland, Australia; 2 Menzies Centre for Health Policy and the Australian Prevention Partnership Centre, University of Sydney, Ultimo, New South Wales, Australia; University of Auckland, NEW ZEALAND

## Abstract

**Background:**

Middle ear disease (otitis media) is endemic among Aboriginal and Torres Strait Islander children in Australia and represents an important cause of hearing loss. The disease is the result of a mix of biological, environmental and host risk factors that interact in complex, non-linear ways along a dynamic continuum. As such, it is generally recognised that a holistic, systems approach is required to reverse the high rates of otitis media in Aboriginal and Torres Strait Islander children. The objective of this paper is to examine the alignment between efforts designed to address otitis media in Aboriginal and Torres Strait Islander children in Queensland, Australia and core concepts of systems thinking. This paper’s overall purpose is to identify which combination of activities, and at which level, hold the potential to facilitate systems changes to better support ear health among Aboriginal and Torres Strait Islander children.

**Methods:**

We began with a review of documents identified in consultation with stakeholders and an online search. In addition, key informants were invited to participate in an online survey and a face-to-face or phone interview. Qualitative interviews using a semi-structured interview guide were used to explore survey responses in more depth. We also undertook interviews at the community level to elicit a diverse range of views. Ideas, statements or activities reported in the documents and interviews as being performed under the Intervention Level Framework were identified using qualitative thematic and content analysis. A quantitative descriptive analysis was also undertaken, whereby data was extracted into an Excel spreadsheet and coded under the relevant strategic directions and performance indicators of the Framework. Subsequently, we coded activities against the five-level intervention framework developed by Malhi and colleagues, that is: 1) paradigm; 2) goals; 3) system structure; 4) feedback and delays; and 5) structural elements.

**Results:**

Overall, twenty documents were reviewed. We examined surveys and interviews with six key informants. Twenty-four individual and 3 group interviews were conducted across central and community level informants. One hundred and four items were coded from the 20 documents and 156 items from interview data. For both data sets, the majority of activities were coded at the structural elements level. The results suggested three key areas where further work is needed to drive sustained improvements: 1) build the governance structures needed for paradigm shift to achieve a multi-sectoral approach; 2) develop shared system level goals; 3) develop system-wide feedback processes.

**Conclusions:**

Sustained progress in improving ear health within Aboriginal and Torres Strait Islander children requires a holistic, system-wide approach. To advance such work, governance structures for multi-sectoral collaboration including the development of joint goals and monitoring and feedback are required. Intervening at these higher leverage points could have a profound effect on persistent public health issues.

## Background

Middle ear disease (otitis media) is endemic among Aboriginal and Torres Strait Islander children in Australia. In many Aboriginal and Torres Strait Islander children, an episode of acute otitis media (AOM) is a catalyst for chronic suppurative otitis media (CSOM), a chronic discharge through a tympanic membrane perforation and a cause of hearing loss [1–3]. Among Aboriginal and Torres Strait Islander populations the prevalence of CSOM is over 4%, exceeding the World Health Organization’s threshold for a major public health problem -[3]. In the worst affected communities, perforation of the tympanic membrane has been reported in more than 50% of children, higher than in any other part of the world [1]. Hearing impairment due to otitis media (OM) is generally conductive in nature, mild to moderate in degree and may be fluctuating or persistent [3–5]and can have an enduring impact on quality of life [6] as well as education and future employment opportunities [1, 2, 7, 8].

The Australian Public Service Commission acknowledges that persistently high levels of OM in Aboriginal and Torres Strait Islander children represents a “wicked” problem [9]. “Wicked” problems are typically socially rather than technically complex, with no single solution and require multi-agency cooperation and coordination to achieve system-wide changes [9]. See Box 1 for some of the main contributing biological, environmental and host factors that contribute to OM in this population.

**Box 1. Biological, environmental and host factors that contribute to otitis media in Aboriginal and Torres Strait Islander children**

**Biological risk factors**: Respiratory bacterial and viral pathogens enter the Eustachian tube from the nasopharynx to the middle ear, causing accumulation of fluid and perforated tympanic membrane [10, 11]

**Environmental risk factors**: season; respiratory viral infection; exposure to other children with otitis media; exposure to tobacco smoke; early introduction of infant formula; use of pacifier; overcrowding in houses; swimming in unclean water; poor access to adequate hygiene and sanitation [11–14]

**Host risk factors**: premature birth, young age; innate immune system; antibody deficiencies [15, 16]

Increasingly, systems thinking has been proposed as a way to understand and tackle complex public health issues such as obesity [17–19], tobacco control [20], antibiotic resistance [21], and the social determinants of health [22]. Malhi and colleagues’ 5-intervention-level framework (ILF) is a tool based on systems thinking that can assist in analysing the potential effectiveness of policy and programme initiatives [23].

In this paper, as part of a larger evaluation, we use the ILF to evaluate activities conducted under the strategic Framework “Deadly Ears, Deadly Kids, Deadly Communities” (DEDKDC) (from this point on known as the “Framework”). The Framework was developed to address OM in Aboriginal and Torres Strait Islander children in Queensland, Australia. To contextualise the paper, we begin by providing a brief description of the Framework, followed by a short introduction to the concepts of systems thinking.

### The deadly ears deadly kids deadly communities Framework

In 2007, the Council of Australian Governments (COAG) agreed to work with Aboriginal and Torres Strait Islander populations to “close the gap” on Indigenous disadvantage. In Queensland, this was reflected in the state Governments plan “Making tracks: a state-wide plan towards addressing the gap in health outcomes for Indigenous Queenslanders 2009–2013”. The Framework aligned with this state-wide plan, and aimed to reduce OM and its impacts in Aboriginal and Torres Strait Islander children [5].

The development and implementation of the Framework was guided by an Interagency Steering Committee which consisted of representatives from Aboriginal and Torres Strait Islander communities; Australian Hearing; Australian Government Department of Health and Ageing; Office for Aboriginal and Torres Strait Islander Health; Australian Government Department of Health and Ageing; Office of Hearing Services; Queensland Aboriginal and Torres Strait Islander Health Council; Queensland Government: Department of Education and Training; Queensland Government: Office of Early Childhood Education and Care; and Queensland Health. Committee members were involved in the development, implementation and monitoring of the Framework. Under the Framework, Queensland Health also managed the Deadly Ears programme, which provided support and training to service providers and mobile ear, nose and throat (ENT) services in a limited number of communities.

The Framework recognised that the persistently high levels of OM in Aboriginal and Torres Strait Islander children was the result of a complex interaction of environmental and host, biological factors and the social determinants of health (refer to Box 1). As such, a variety of multi-sectoral activities at different levels of the system (e.g. central level policy-makers, service providers and local communities) were proposed. These activities were underpinned by five principles and assumptions, three goals and two strategic directions (Box 2). For each of the goals, measurable targets that aligned with the two strategic directions were articulated.

**Box 2. Principles, assumptions, goals and strategic directions of the Framework**

**Principles and assumptions**

1. Evidenced-based practice and policy
2. Community engagement in health promotion
3. Access to primary health care
4. Access to mainstream services
5. A population-based approach

**Goals**

1. Reduce the incidence and impact of Otitis Media and Conductive Hearing Loss in Aboriginal and Torres Strait Islander children living in Queensland
2. Improve the coordination and integration of culturally appropriate and evidence-base sustainable ear health services across the care continuum for Aboriginal and Torres Strait Islander children living in Queensland
3. Integrate ear health services into primary and public health care services/approaches to Aboriginal and Torres Strait Islander child, maternal and family health

**Strategic directions**

1. Develop and enhance interagency services that support a culturally appropriate and evidence-based approach to the management of Otitis Media in the areas of:
  - prevention
  - screening, surveillance and diagnosis
  - treatment, care and support.
2. Develop system enablers that support a culturally appropriate and evidence-based approach to the management of Otitis Media across the care continuum in the areas of:
  - partnerships
  - workforce development
  - information and knowledge

## Systems thinking

Meadows, a key thinker in systems thinking, defines a system as:

*A set of elements or parts that is coherently organized and interconnected in a pattern or structure that produces a characteristic set of behaviours, often classified as its “function” or “purpose””*.[24]

Systems thinking is an iterative process which takes a broad, integrated perspective examining the linkages and interactivities among the systems elements [25–30],,]. It involves looking at issues holistically, identifying interrelationships, patterns of change and the context in which they occur, rather than snapshots of change at a particular point in time [30–33]. Actors in the system are seen to be interrelated, each acting according to their individual strategies or routines while at the same time, acting and reacting to what others in the system are doing (for example, public servants, managers, local service providers, teachers, nurses, community leaders and intended programme recipients) [33, 34]. Partly because of this, changes in the system are non-linear, with small changes sometimes having a large effect, and large changes potentially having limited impact [20, 25, 32, 33]. Understanding leverage points within a system and monitoring feedback loops to understand how the system is working can help explain how shifts in one element of a system produce change in another element [35]. Each system exists within, and interacts with, a hierarchy of systems. For example, within a health service there are several sub-systems, such as, clinical services, information systems, finance, and administration departments. Box 3 summarises the characteristics of systems thinking.

**Box 3. Characteristics of systems thinking based on Meadows [24]**

- Views a system as a set of distinct parts that act together to form something more complex
- Focuses on the whole, rather than the separate parts of a system
- Sets system level goals
- Identifies interrelationships and patterns of change over time
- Recognises the importance of feedback loops in providing information how the system is working
- Looks for leverage points where a small amount of change can cause a large change in the system
- Highlights the importance of context in which an action is taken to understand outcomes
- Identifies non-linear relationships
- Is purposeful, seeking to identify where to intervene in a system to influence system change

Utilising constructs of the ILF, the objective of this paper is to examine the alignment between efforts designed to address otitis media in Aboriginal and Torres Strait Islander children in Queensland, Australia and core concepts of system thinking. The overall purpose is to identify potential strategies and levels of intervention to facilitate systems changes to better support ear health among Aboriginal and Torres Strait Islander children.

### Evaluation design

The evaluation design uses qualitatively driven methodologies including document review and semi-structured interviews with key informants. This design enabled the triangulation of data from different sources to support a broader understanding of findings in context.

### Methods

Mahli and colleagues’ ILF [23] draws on the work of Meadows’ 12 “places to intervene” in a system [24]. The ILF has five mutually exclusive levels of intervention: 1) paradigm; 2) goals; 3) system structure; 4) feedback and delays; and 5) structural elements (refer to Table 1). It has been used to analyse activities contained in policy and strategic documents to improve food systems [23] and obesity [27] and has been shown to provide way of sorting activities in a reproducible manner against the five levels [23].

**Table 1:** The five levels of the Intervention Level Framework as described by Malhi et al. [23].

Level	Description
Paradigm	Unstated assumption under which a system operate A system's deepest belief and the source of system goals, information flows, feedback It is very difficult to intervene at this level, but it can be very effective
Goals	What the system is trying to achieve—the drivers of the system, with everything below working towards their achievement Activities at this level focus or change the aim of the system
System structure	Enhancing connections across most of the system as a whole Activities at this level will shift the system structure by changing system linkages or incorporating novel elements All of the elements that make up the system as a whole including the various sub-systems, actors and their interactivities Includes the “rules of the game” that governs the system and controls information flows
Knowledge, feedback and delays	Allows the system to regulate itself by providing information about the outcome of different activities back to the source of the activities Can be simple and direct or involve multiple variables Can include monitoring and evaluation
Structural elements	Affect subsystems, actors, and the physical structure of the system Easiest level at which to intervene Many activities at this level are usually required to create system-wide change

## Review of the Framework documents

First, we reviewed the Framework document for background information in order to identify the underlying principles of the Framework itself, its goals, planned activities and what it attempted to achieve. Other documents were identified in discussions with stakeholders and through an online search. A matrix of available documents was developed, noting the publication title, type (e.g. annual report, evaluation), author, and year of publication. Retrieved documents included programme field reports, evaluations, annual reports, relevant state policies and procedures for the Framework’s time period (2009–2013). Broad national or state level policy or strategy documents that mentioned the Framework as part of an overall approach were excluded given our focus on activities undertaken as part of the Framework. In addition, documents that mentioned OM in Aboriginal and Torres Strait Islander children but did not propose specific activities (e.g. in The Chief Health Officer’s Report for Queensland) were excluded (n = 7).

Three researchers (JD, LS and LV) read all of the documents. Ideas, statements or activities undertaken as part of the Framework were identified and catalogued in a matrix initially sorted against the relevant strategic directions and performance indicators in the Framework. To capture a broad picture of the types of activities against each level of system function, one author (JD), subsequently sorted the statements and compiled them into a preliminary list based on the ILF definitions. These were checked by a second researcher (LS) and where there were differences, the documents were reviewed again to seek clarification. Following Malhi et al similar ideas were consolidated and grouped together to reduce redundancy [23].

Action statements that related to field trip reports, needs analysis, evaluations, development of an ear, nose and throat (ENT) database, and presentations were coded as feedback. Where there was insufficient detail in the available documents to determine the number of times an action had occurred (e.g. the use of specific equipment in community health centres) the statement was recorded once. This means that some activities may be underreported in our analysis. Further, as our focus was on activities, we did not code results. For example, we did not include statements such as “enhanced availability of quality post-ENT clinical and surgery data” in the coding.

## Surveys and interviews

### Sampling

All steering committee members (n = 14) were invited to participate. A mix of individuals from two communities with knowledge and experience of the Framework were also invited to participate. These participants included community leaders, educators, healthcare workers and other service providers involved in work with Aboriginal and Torres Strait Islander children and families allowing us to gain a broad range of perspectives regarding implementation of the Framework.

### Data collection

Steering Committee members were invited to participate in an online survey and a face-to-face or phone interview. The survey utilized closed questions with the option of providing additional comments after each question. Survey questions related to alignment of the Framework goals with organisational goals, perceived cost/benefits of participation in the Steering Committee in terms of achieving organisational goals, Framework effectiveness, and the monitoring of activities performed under the auspices of the Framework.

Follow up interviews with Steering Committee members allowed for a more in-depth examination of issues raised in the survey. Interviews at the community level used a semi-structured interview guide with questions tailored to the different stakeholders. In general, questions pertained to community and organisational goals, perspectives of OM in the community and community participation in activities designed to prevent and treat OM and knowledge of the Framework. The use of a guide allowed us to focus on the topic while at the same time allowing participants to talk about the Framework (and/or ear and hearing health and services) in their own words, concentrating on the issues that they felt were important. This provided the interviewer with the flexibility to follow-up, clarify participant ideas and adapt interview questions as the study progressed and new insights were gained. The majority of interviews were conducted in person by a member of the research team with an additional researcher taking notes (JD, LS or LV). Interviews were recorded with informed consent.

### Data analysis

Interviews were professionally transcribed, and reviewed by the interviewers (JD, LS, LV) prior to analysis to ensure accuracy. The data was entered into the qualitative software management tool Nvivo version 10 (QSR International) [36]. All transcripts were read independently by three members of the evaluation team (JD, LS and LV) to identify initial codes. The data was then coded iteratively in Nvivo using qualitative thematic and content analysis, with the evaluators seeking and coding recurring patterns following a collaboratively agreed upon coding system based on the evaluation questions.

A quantitative descriptive analysis was also undertaken, whereby data was extracted into an Excel spreadsheet and coded under the relevant strategic directions and performance indicators of the Framework. Subsequently, activities were coded against the five-level intervention framework developed by Malhi and colleagues, as described previously [23].

Activities undertaken by interviewees or their organisations that were not directly related to activities under the Framework were not coded, nor were outcomes coded. Where participants mentioned the same activity in an interview the statement was recorded once.

### Ethics

Full ethical approval was obtained from the Children’s Health Queensland, Hospital and Health Services Human Research Ethics Committee (ref: HREC/14/QRCH/113) and The University of Queensland’s Medical Research Ethics Committee (ref: 2014000963). All participants provided their written informed consent to participate in the study.

### Results

One hundred and four items were coded from twenty documents. The twenty documents were: the Framework (n = 1); annual reports (n = 7); a community-based survey (n = 1); an evaluation report (n = 1); field reports (n = 7); Memoranda of Understanding (n = 2); and model of care document (n = 1). Table 2 provides examples of the type of activities contained in the reviewed documents as coded under each of the five leverage points in the ILF.

**Table 2:** Examples of activities identified in the Framework documents review and how they were coded at the different levels of the ILF.

Level	Document review example activities
Paradigm	Evidenced-based practice and policy Community engagement in health promotion Access to primary health care Access to mainstream services; and a population based approach.
Goals	Three health related goals to: Reduce the incidence and impact of Otitis Media and Conductive Hearing Loss in Aboriginal and Torres Strait Islander children living in Queensland Improve the coordination and integration of culturally appropriate and evidence-based sustainable ear health services across the care continuum for Aboriginal and Torres Strait Islander children living in Queensland Integrate ear health services into primary and public health care services / approaches to Aboriginal and Torres Strait Islander child, maternal and family health
System structure	Memoranda of Understanding with training providers and health services Advocacy for resourcing options and models of care for allied health in Aboriginal & Torres Strait Islander communities Ear disease and its impacts are included in the draft Blueprint for better health outcomes for Aboriginal and Torres Strait Islander people in Queensland
Knowledge, feedback and delays	Database containing information on one individual programme activities Workforce survey and needs analysis Survey of classroom teachers using sound field systems Cross-sectional assessment of hearing in school aged children (aged 4–9 years) Conference presentations and peer reviewed publication Annual reports (2009–2013) Evaluations, social marketing and health promotion campaigns
Structural elements	Development of educational resources for health workers, maternal and child care workers and educators including on-line courses for early educators
	Training workshops for Child Health Nurses, Clinical Facilitators and qualified health workers
	Ear health and strategies included in Cert IV Training and Evaluation for Early Child Educators
	Provision of Sound Amplification Systems to schools and classroom acoustic modifications
	Funding provided to address financial disincentives experienced by health professionals providing outreach services to provide multidisciplinary outreach services (GPs, nurses, medical specialists, speech therapists, audiologists)
	Provision and maintenance of equipment (e.g. otoscope, tympanometer and audiometer)
	Recommendations for Clinical Care Guidelines on the Management of Otitis Media in Aboriginal and Torres Strait Islander Populations and Personal Health Record updated and promoted
	Ear health surgical services and audiological checks Social marketing strategy including development and dissemination of DVD and development of a central portal for information, publications on ear and hearing health resources and healthy lifestyle messages and Otitis Media info sheet
	Forum held with key clinicians, managers and academics to develop a primary prevention strategy
	Provision of reporting templates for performance reporting

Six out of fourteen Steering Committee members returned their completed survey forms and were interviewed. In addition, three interviews (one conducted with two participants) were held with Deadly Ears Programme staff members to discuss their sector work. At the community level, 18 individual interviews and 3 group interviews were undertaken. They included a government worker (n = 1), primary school classroom teachers (n = 5, including 1 special education teacher), senior school management (n = 4), a nursing director (n = 1), child health nurses (n = 2), a local council member (n = 1), community healthcare workers (n = 4), community child care workers (n = 5), local social workers (n = 2) and community elders (n = 2).

**Fig 1:**
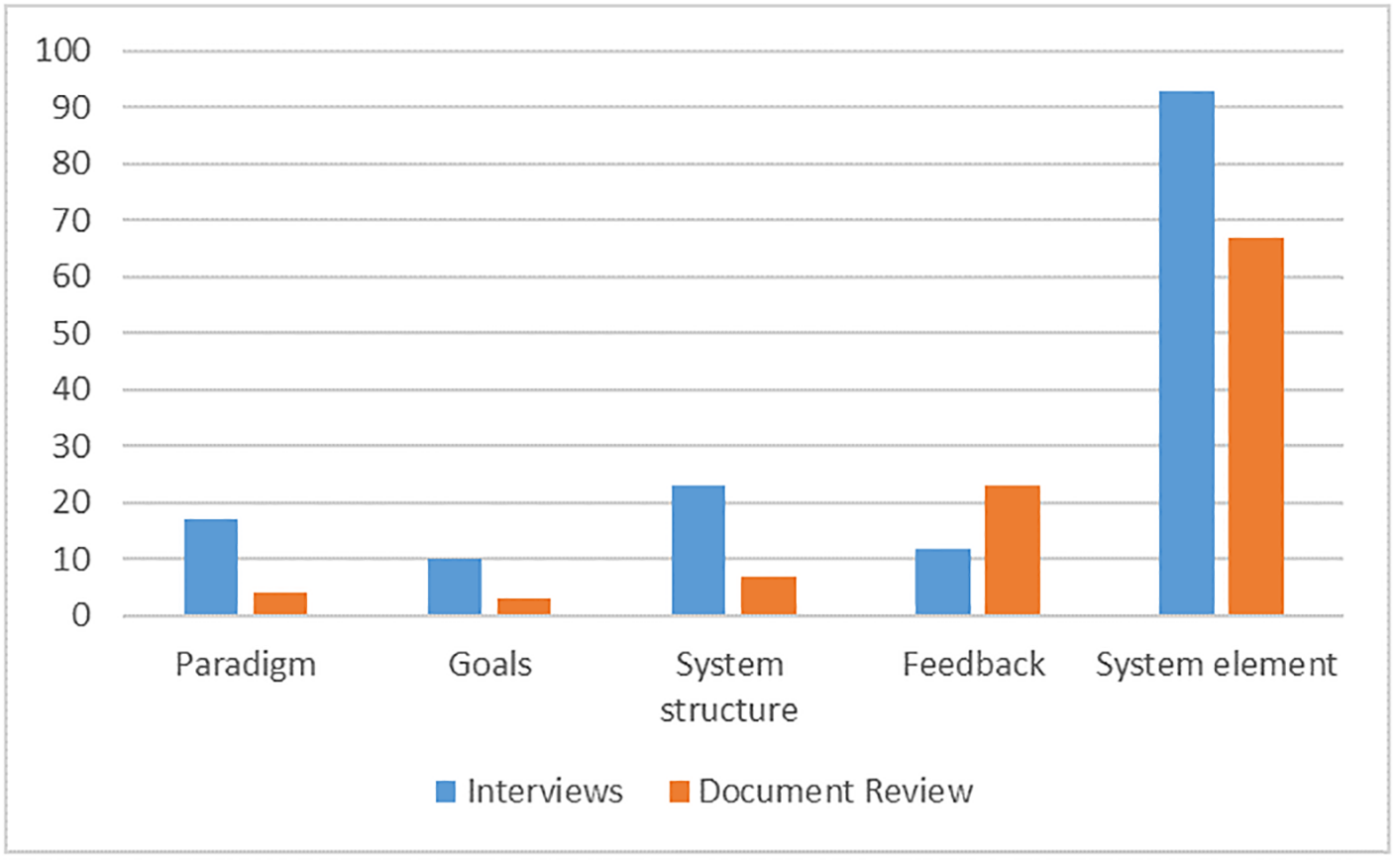
Number of interventions coded at each level of ILF in the document review and the interviews.

We coded 156 items in the interview data. Table 3 provides examples of the type of activities reported in interviews coded under the ILF leverage points. Fig 1 presents the relative distribution of activities coded under the ILF based on the document review and the qualitative interviews. It shows that for both data sets, the majority of activities were coded at the structural elements level (66%, n = 67 documents review and 60%, n = 93 in the interviews).

**Table 3:** Examples of activities identified in the interviews and how they were coded at the different levels of the ILF.

Level	Example activities in the interview data
Paradigm	Making sure that all of that is up to date with current best practice processes as well This needs to be implemented because it’s evidence-based resource Community engagement Population-based approach Primary prevention Community engagement A coordinated approach across sectors
Goals	Aligning with the national quality framework Working with education, early childhood, healthcare workers and allied health Joint goals
System structure	Memorandums of Understanding Working to integrate ear health into the curriculum at university level Aligning ear health with national quality frameworks for early education Supporting the curriculum of early educators and teachers Being on an advisory group to develop a strategy for early years training
Knowledge, feedback and delays	Feedback on resources needed (e.g. posters) Conference presentation Feedback within communities Feedback on activities undertaken provided to Steering Committee Acting as an information bridge across sectors Clinical data
Structural elements	Developing online professional development and support for teachers who teach in schools Delivering presentations at the teachers, nurses and therapists forums Provision of training Provision of Sound Amplification Systems to schools and classroom acoustic modifications Social marketing Informal discussion with teachers and supporting early child educators within the communities Supporting guidelines for child health checks to include ear and hearing health checks Practical workforce clinical development Provision of clinical guidelines Sharing information with healthcare workers and educators that can be passed onto the community Developing scripts that maternal and child healthcare workers can use with mothers Working with play groups in the communities Delivery of ear nose and throat clinical services Clinical student placements

The remainder of this section presents the results of the documents review and the qualitative interviews under the five headings of the ILF.

## Paradigm level results

### Document review

The Framework was underpinned by five principles and assumptions (Box 2). It recognised the need for a holistic, multi-sectoral approach to improving ear health in Aboriginal and Torres Strait Islander children. The principles however, largely related to public health and did not emphasise multi-sectoral collaboration (Box 2). This was also reflected in the annual reports which showed that most of the activities undertaken at this level reflected principles of evidence-based clinical practice.

### Interviews

As seen in Table 3, the interviews included more paradigm level statements than the document review did (n = 17 in interview data and 4 in the document review). Statements at this level were from Steering Committee or central level staff and broadly reflected the content contained in the reviewed documents (Table 3). Evidence-based policy and practice was most commonly mentioned at this level (59%, n = 10). The following interview extracts are illustrative of this:

[we are] very much working to that policy and evidence based Framework

*(C_006)*.

..*it also just reiterates best practice approaches for all the teachers so that it doesn’t matter if you have a classroom full of kids with conductive hearing loss or you have a classroom full of kids who don’t have any hearing loss, it’s actually still all going to make a difference to the kids’ learning and listening environments regardless*.

(W_006)

Interviewees recognised the need for a multi-sectoral approach but very few (12%, n = 2) activities were coded at this level. Both inter-department and inter-agency collaboration was reported to be difficult to implement in practice. As one Steering Committee member reported, “we’ve not ever got to that stage” (C_006). Another person explained:

.. *we’re still very much at work in our own little corners at the moment and it’s very hard, even for two inter-agencies, to work together. You know, it’s just really, really hard*.

(I_012)

The reasons for limited multi-sectoral activities included lack of governance mechanisms and high-level leadership to facilitate this work. The following interview excerpt illustrate this point:

*it needs someone [with] some clout and a chance of actually changing and mandating some of that work………there [also] needs to be better engagement and involvement that actually drives people making the commitment and seeing an integrated commitment rather than a–‘this is my bureaucratic responsibility, I’ll do that bit and hope to God the rest works’*.

(C_003)

Other barriers included a lack of understanding of different department processes, funding constraints and differing priorities. Not having a shared language was also mentioned and something to be overcome by “*building narrative*, *our story*, *why this is important for Aboriginal and Torres Strait Islander children*” (W_006) and using new language, as one person explained:

.. *they teach us a lot about their lingo and so that when we are delivering things to teachers it’s not coming from a health perspective, it’s coming from an education perspective and making sure that the language that we use is familiar. They use things, like, I would say, “Adjustments,” and they would say, “Differentiated instruction.” And yeah, we would say, “Acoustic environments,” they would say, “In-classroom listening.”*

(W_006)

### Goal level results

#### Document review

The Framework document described three health orientated goals (see Box 2). Progress against these goals was recorded in annual reports in the form of outputs (e.g. number of activities) with reports publically available via a website. No outcome data (i.e. data on the degree to which activities were having an effect on the target population’s practices) was available in the reviewed documents.

#### Interviews

At the goal level statements were mainly made by Steering Committee members and participants working at the central level. Whereas the goals in the document review focused on health, during interviews, participants talked about the need for both health and multi-sectoral goals as the following quotes illustrate:

we’re trying to take a systematic approach rather than helping with the direct service-specific issue

(W_001)

So it’s just about having a coordinated approach

(C_005)

[we aim to] meet those really fundamental core areas of prevention, screenings, advanced diagnosis, treatments, support

(W_002)

So our long term goal is to really influence how other clinicians in mainstream services do their practice and do their business and so to be providing information into that research base

(W_003)

In four of the six interviews with Steering Committee members, the lack of shared Framework goals was reported as an impediment to multi-sector collaboration. As one Steering Committee member explained:

We don’t have joint goals or joint priorities that we could be working towards. Because essentially we are all working to the same outcome around close the gap and improving health outcomes for the population but it kind of, how we get there, or how we are going, it’s all different and at different paces, not necessarily in the same lane

(W_006)

Over time, Steering Committee meetings also shifted from being multi-sectoral, to individual sector specific meetings. Not all of the Committee members (n = 3) felt that this shift was useful however, recognising that talking together could act as an impetus for change:

I would like to see that at least once a year, maybe we all get together, because, just having those opportunities to talk to people, to get an idea of the systems and the barriers and realities of what people in other sectors…—when you actually sit there and get that opportunity to talk, your ideas are maybe modified

(C_005)

### System structure level results

#### Document review

The document review resulted in 14 activities being coded at the system structure level. These included efforts to build collaborations across subsystems and partnerships, including through Memorandum of Understandings (MoU). Other activities coded at the system structure level included negotiating for multidisciplinary health care teams and liaising with education, allied health and public health. Clinical guidelines were developed and disseminated to clinicians but no information was available in the reviewed documents on how these guidelines were being applied in practice.

#### Interviews

Analysis of the qualitative data resulted in 23 activities being coded at the system structure level. Of these, six stated the need for more strategic formal partnerships, rather than relying on ad-hoc, personal connections. One person explained:

we need to have a look at what we need to happen in order for our end goal of, you know, ears and hearing being improved and what are those really key activities that stem from that and who is the best person that we need to partner to get that activity completed in a sustainable and systematic way that’s effective, not ad hoc. …… we really need that really systematic approach, so you're looking at the systems rather than the people

(W_002)

Few participants felt that the Framework had contributed to developing cross-sectoral collaboration. A senior community representative working for an organisation where there was a MoU in place reported that:

.. *even at those [strategic] meetings we don’t talk about anything there [to do] with ear health–and I think that probably reflects the fact that we don’t have any contact, really, with the [people] from Brisbane*

(I_010)

### Knowledge, feedback and delays results

#### Document review

In the document review, we coded 6% (n = 25) of activities as feedback. Almost all (n = 24) came from the annual reports and related to Steering Committee members’ activities conducted under the auspices of the Framework. Other feedback mechanisms included evaluation of processes such as the use of new instruments in clinical settings and the user’s self-reported confidence in applying these. Field trip reports, mainly undertaken by the Deadly Ears programme in the communities where it worked, were another form of feedback. Feedback in these reports were primarily qualitative, focussed on specific activities and were used to inform programme planning at the individual-community level. Other feedback included dissemination of information through meetings and presentations.

Information on the incidence of CSOM came from the Deadly Ears ENT clinics. This data indicated that the presentations of CSOM in both 0–4 and 4–14 year olds from 2009–13 had reduced, but it was not possible to assess reductions in the incidence of CSOM state-wide due to the lack of population level data.

#### Interviews

In the interview data we coded 8 percent (n = 12) activities at the feedback level. Most of the reported activities related either to sharing information opportunistically or within specific teams (n = 5) and related to activities undertaken at the system element level as the following quotes illustrate:

.. *someone in community—from the team, just say, for instance, notices that or comes with that—finds out that information that child health checks are not—the ear check isn’t complete or whatever. So that’s when that information can come back to meetings and then go to the sector guys, the maternal and health*

(W_001)

*[we had] a couple of posters which we did, implemented in that community. Then opportunistically we shared with a support agency and they said, “Oh, that would be really great, we've had lots of requests from our other services. It would be really good if we could flesh it out and make it more bigger, have some training attached to that.” And then we've brought it back to here, to the sector meeting, and it’s coincided with some other feedback that we've received at other opportunities at a sector level as well*.

(W_001)

There was limited evidence however, of feedback being used strategically to the right place within the system to create change as the following interview excerpts help to highlight:

*I don’t know that… (getting) the results of studies into the hands of the practitioners on the ground so that they can actually do it really well*.

(C_003)

.. *it’s not actually the community strategy stuff that’s going up to the Steering Committee*

(W_003)

Getting feedback on changes in the incidence and prevalence of OM was reported to be nearly impossible due to the lack of a standardised, state-wide system:

Different data systems, different definitions, different processes, different reporting techniques, different denominators and numerators. It's meaningless

(C_001)

There was also limited feedback on how practitioners implemented evidence-based practice within the context of their day-to-day work. As one person explained:

They [evidence-based best practice] are spelt out in the clinical guidelines, but the systems that are in place don’t lend themselves for those clinical guidelines to be implemented easily

(C_005)

While near universal school based screening is not recommended as a preventative action, senior educations staff and teachers (n = 9) saw school screening as providing important feedback on individual children, according to one person:

*The information that I am able to feed from those [bi-annual] screenings back to teachers around individual children who may be having some hearing loss at any particular point in time, or they might have a hearing loss in one ear, it allows the teacher to think strategically about how they might position that child in their classrooms*.

(I_015)

Few of the respondents at the community level were aware of the activities undertaken as part of the Framework and just over half (n = 12) felt that they would like more feedback on overall Framework performance. None of the respondents at the community level reported being aware of the annual reports.

### Structural elements level results

#### Document review

In the document review, the majority of the activities were coded at the level of structural elements (n = 67). Many of the activities (n = 27, 40%) at the structural level were intended to increase the technical capacity of teachers and primary healthcare providers through face-to-face and on-line training. Other activities at this level included social marketing; health promotion; workshops; providing clinical equipment (e.g. otoscope, tympanometer and audiometer) to health workers; and improving the acoustic classroom environment to support hearing-impaired students.

#### Interviews

As in the document review, the majority of the activities reported in the interviews were coded at the level of structural elements (n = 93). Activities mentioned largely reflected those included in the document review and included social marketing; classroom acoustic modification; and training for teachers and health workers.

## Discussion

In this paper we applied the 5-level ILF developed by Malhi, et al. [23] to examine the alignment between strategies used to address OM in Aboriginal and Torres Strait Islander children under the Framework and core concepts of systems thinking. Application of the ILF helped to identify three key areas where further work is needed to drive a sustained reduction in OM within Aboriginal and Torres Strait Islander children: 1) building the governance structures needed for paradigm shift to achieve a multi-sectoral approach; 2) developing shared system level goals; 3) developing system-wide feedback processes. The ILF also helped us to identify that most activities were taking place at the system element level which, while necessary, is often the least effective part of the system to intervene for sustained change [37].

At the paradigm level, the Framework recognised the need for multi-sectoral action, yet the extent to which this was achieved was limited. This helps to illustrate that even where the need for multi-sectoral action is recognised, it can fail to take hold [38–40]. To facilitate multi-sectoral action interventions that specifically aim to change “the rules” of a system may be needed. This could include for example, redefining system boundaries to allow multi-sectoral work and developing the governance structures to support this change [24, 40]. The collection and reporting of information, implicit in multi-sector efforts can also act as an effective leverage point as long as information is presented in a compelling form and flows to the right place within the system [24, 40].

In any intervention, goal setting is important because it determines what gets done [27, 38]. The Framework goals were relatively narrow and to drive whole system change, goals need to be truly multi-sectoral and relevant to the entire system [22, 39–42]. An example of a whole system is: “To promote healthy environments for Aboriginal and Torres Strait Islander children to grow and thrive”.

Feedback plays a central role in systems thinking and in enabling systems to achieve their goals [23, 29, 35]. Feedback loops can be balancing or reinforcing [24]. For example, due to the infectious nature of OM, the higher the prevalence of OM in the community the higher the incidence of new cases is likely to be creating a reinforcing feedback loop [24]. Balancing this feedback loop requires preventative efforts, early detection and initiation and completion of treatment. Activities conducted under the Framework however, while important, did not address the broader determinants of OM, making the balancing effect too small in comparison to the factors it was trying to counter, limiting the potential for sustained change [24, 40]. Furthermore, feedback did not reach key actors across the different elements of the system minimising the potential for these actors to take corrective actions. The focus on individual organisational level outputs also potentially undermined collaborative, multi-sectoral approach engagement and analysis [24, 31, 38, 40, 42].

The system element constituted the majority of activities, including, training health and education staff, providing ENT services and classroom acoustic modifications. This is the level at which activities are most likely to have a direct impact on individuals [27]. While necessary, this is often a “weak” leverage point with changes often “washing out” as the system returns to the status quo [23, 25, 35, 41].

### Implications for policy and practice and strategies to facilitate system change

A stronger understanding of systems thinking may assist policy makers, programme managers and practitioners to better grasp the complexities of OM in Aboriginal and Torres Strait Islander communities. Specifically, it can help to identify which combination of activities and at which level, hold the potential to contribute to wider system change. Table 4 outlines some potential strategies that organisations could utilise to facilitate system change.

**Table 4:** Potential strategies to facilitate system change to improve ear health in Aboriginal and Torres Strait Islander children.

Level	Potential strategies
Paradigm	Examine the physical, social, and economic environment and its interactions with diverse stakeholders to collaboratively develop system level goals Repeatedly and consistently challenge assumptions, values and priorities of the different and diverse actors who can contribute to improving ear health to promote change Develop governance, co-financing, and co-monitoring mechanism to facilitate of multi-sectoral activities to address the social determinants of ear health Develop specific guidance on the governance of working multi-sectorally Identify and work with key decision makers and change agents in the different levels of the system (e.g. State, local government, councils and communities)
Goals	Develop whole system goals that all stakeholders can agree to (e.g. State, local government, councils and communities) and across sectors such as health, education and training, early childcare, environment, and Housing and Public Works, Aboriginal and Torres Strait Islander partnerships
System structure	Enhance connections across the system
	Establish and articulate simple rules collaboratively and cross-sectorally
	Include interventions that focus on changing the physical, social, and economic environment to complement individualized approaches
Knowledge, feedback and delays	Build ongoing feedback and monitoring into the system with flexibility to adjust intervention’s based on feedback Minimise delays in feedback and information flows at different level of the system Monitor changes in social and political context and community norms and respond where activities are not having the desired effect (e.g. changes in government and policy, changes in practices in relation to ear health as well as practices such as changes in services and service utilisation) Include process and summative evaluation to understand what works, how and in what contexts and monitor changes in patterns that are indicative of change as outcomes may not be observed in short timeframes (e.g. changes in presentations at primary health care facilities or changes in school attendance Alignment with the Recommendations for Clinical Care Guidelines on the Management of OM in Aboriginal and Torres Strait Islander Populations for surveillance and diagnosis Provide timely, clear and truthful feedback to stakeholders at all levels of the system in ways appropriate for the target population Engage a diverse range of stakeholders at different levels of the system in feedback processes with an emphasis on both qualitative and quantitative feedback (e.g. change in number of clinicians using OM clinical guidelines, changes in levels of behavioural risk factors associated with OM, changes in knowledge and/or skills to prevent and manage OM in service providers and affected population)
Structural elements	Pay attention to context, identifying potential bottlenecks and contextual factors that enable or hinder the required change (e.g. lack of equipment capacity to use it, bottlenecks in delivery of sound field systems, contextual factors that prevent implementation of OM clinical guidelines, or limit teaching practices that support learning for children with poor ear health) Iterative negotiations with stakeholders to understand context and facilitate change processes at the different levels of the system

## Limitations

This evaluation study has several limitations. Despite repeated efforts to reach Steering Committee members, the survey response rate (43%, n = 6) was low and some perspectives may not have been captured in the data. However, there was no disproportionate representation of absent perspectives from the Steering Committee members as all major sectors on the committee were among those that did respond. Another limitation of our evaluation is that the data set from which we extracted the interventions was based mainly on activities included in publically available annual reports, and as such, we may have missed some activities. In addition, while we tried to verify, to the extent possible within the scope of the evaluation, that the reported action had taken place, we were not able to confirm this with all activities. These limitations were potentially mitigated however through triangulation with other Framework documents and stakeholder interviews.

## Conclusion

Sustained progress in improving ear health within Aboriginal and Torres Strait Islander children requires a holistic, system-wide approach. To progress such work, governance structures for multi-sectoral collaboration including the development of joint goals and monitoring and feedback are required. Intervening at these higher leverage points could have a profound effect on persistent public health issues.

## Abbreviations

COAG: Council of Australian Governments
CSOM: Chronic Suppurative Otitis Media
DEDKDC: Deadly ears, Deadly kids, Deadly Communities
ILF: Interventions Level Framework
OM: Otitis Media
TM: Tympanic Membrane
